# The impact of open access mandates on scientific research and technological development in the U.S.

**DOI:** 10.1016/j.isci.2023.107740

**Published:** 2023-08-26

**Authors:** Benedict Probst, Paul M. Lohmann, Andreas Kontoleon, Laura Díaz Anadón

**Affiliations:** 1 Group for Sustainability and Technology, Department of Management, Technology, and Economics, ETH Zurich; 2 El-Erian Institute of Behavioural Economics and Policy, Judge Business School, University of Cambridge, UK; 3 Centre for Environment, Energy, and Natural Resource Governance, Department of Land Economy, University of Cambridge; 4 Harvard Kennedy School, Harvard University, United States

**Keywords:** Social sciences, Research methodology social science

## Abstract

Getting to a net-zero emissions economy requires faster development and diffusion of novel clean energy technologies. We exploit a rare natural experiment to study the impact of an open-access mandate on the diffusion of scientific research into patented technologies. From 2014 onwards, the U.S. Department of Energy (DOE) required its 17 National Laboratories (NLs) to publish all peer-reviewed scientific articles without a paywall. Using data from more than 300,000 scientific publications between 2012 and 2018, we show that scientific articles subject to the mandate were used on average 42% more in patents, despite embargo periods of up to 12 months. We also show that articles subject to the mandate were not cited more frequently by other academic articles. Our findings suggest that the mandate primarily contributed to technological development but has not led to additional academic research. Lastly, we show that small firms were the primary beneficiaries of the increased diffusion of scientific knowledge.

## Highlights

• We examine the impact of the U.S. Department of Energy’s open-access mandate
• Scientific articles subject to the mandate were utilized on average 42% more in patents
• Articles subject to the mandate were not cited more frequently by other academic papers
• Small firms were the primary beneficiaries of the increased knowledge diffusion

## Graphical abstract

**Figure:**
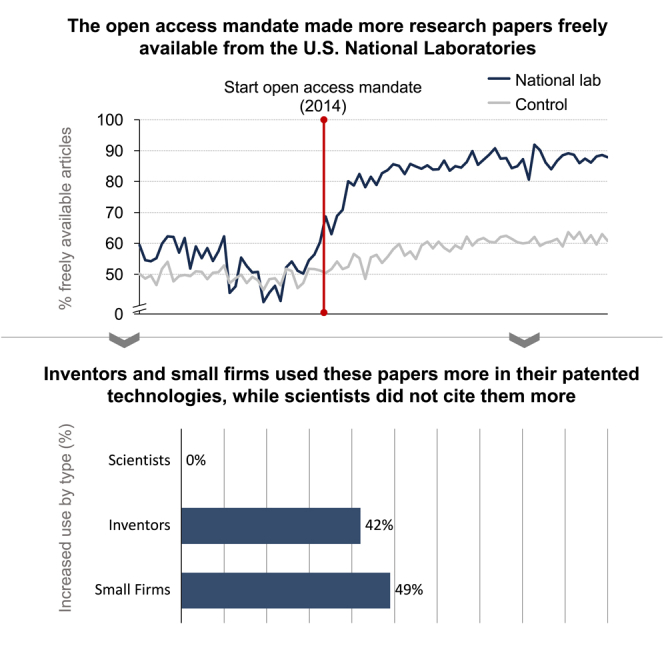

## Introduction

Achieving net-zero emissions by the second half of this century as envisaged by the Paris Agreement^1^ requires—among other things—faster development and diffusion of novel clean energy technologies.^2^ One of the factors that could shape the impact of scientific research on technological development is the extent to which inventors globally have knowledge about and access to those publications. As the predominant model of scientific publishing continues to rely on a subscription-based system, inventors without access to major journals paid by their universities, laboratories, or companies may have difficulties accessing scientific publications.^3^ While the number of open access (OA) journals, publications in paywalled journals published as OA (so-called “hybrid OA”), and articles made available in repositories (so-called “green OA”) has increased substantially over the last decade, the most highly cited academic journals maintain a subscription-based model of publishing.^4^

For inventors, the importance of accessing scientific literature has grown over time as scientific research is playing an increasingly critical role in shaping technological progress. The share of U.S. patents reliant on federally funded science has increased steadily over the last century, with nearly one-third of patented inventions in the United States (U.S.) citing federally funded science since the 2000s. In the U.S., federal agencies play a key role in funding and conducting scientific research like the federal executive departments (e.g., defense, health, and human services), the National Science Foundation, and NASA.^5^ In the energy domain, the Department of Energy (DOE) plays a key role in driving scientific and technological progress. For instance, the DOE directly supported around 4% of all U.S. inventions in 2017.^6^ The DOE’s National Labs (NLs) are considered the “crown jewels” of innovation and form a critical part of the U.S. energy innovation sphere. Yet, reviews of the NL’s performance have highlighted several areas of weak organizational design, specifically on accelerating the outward diffusion of scientific advances to industry.^7^

Over the past five years, several universities and other public research institutions ranging from the University of California to the Max Planck Institute have voiced increasing discontent with the subscription-based system adopted by most academic journals. Reasons for this resistance include perceptions of unfair pricing and the assertion that the results of publicly funded science should be available to the public.^8^ The most wide-ranging OA initiative unveiled in 2018 is dubbed “Plan-S”, which requires scientists receiving funding from European and other research organizations controlling EUR 33 billion in annual funding to make their research directly available upon publication from 2021 onward, at least in a repository.^9^^,^^10^ Recently, the U.S. has followed suit—based on guidance by the White House Office of Science and Technology Policy^11^—by announcing that government-funded science needs to be freely available upon publication from 2026 onwards.^12^

As scientific journals provide a central diffusion pathway of scientific knowledge to firms,^13^^,^^14^^,^^15^^,^^16^ understanding the effect of journal access on technological development is critical. Yet, the relationship between the degree of accessibility of a scientific publication and its subsequent impact on technological applications—in other words, commercial and wider economic impact—proxied by patents is, however, unclear (except for Bryan and Ozcan (2021) who study the effect of OA on inventions in the biomedical sector). Understanding the commercialization of energy R&D is particularly important as the research needs to ultimately be applied and further developed by companies (in contrast to military R&D where the government is both the producer and “purchaser” of novel technologies).

We exploit a rare natural experiment to study the impact of the increased availability of scientific research on the diffusion of scientific knowledge into patented technologies. The U.S. DOE mandated that all scientific, peer-reviewed articles resulting from its 17 NLs must be made available without a paywall within a year if published on or after the 1^st^ of October 2014.

The mandate allows us to study three interrelated questions. First, we analyze how NL researchers changed their publication strategy based on the mandate. If researchers choose to deposit accepted manuscripts in free repositories, many journals impose an embargo period, which is the time lag before it can be made publicly available in a repository. In our sample, 11 out of 16 paywalled journals had embargo periods of at least 6 months, thereby increasing access hurdles to the diffusion of research. If researchers pay for gold open access, this could divert funds away from key research activities as open access fees of 10,000 USD per article are not unusual.

Second, we analyze whether treated articles (i.e., those emanating from the NLs subject to the OA mandate) were used more in other scientific articles and patented technologies. We investigate whether articles subject to the mandate were cited more by other academic research and patents (we use the latter as a proxy for technological development). Lastly, we investigate whether the mandate primarily helped those firms with historically low access to scientific research (e.g., small firms).

Exploring whether small firms are affected by the mandate is critical for several reasons. Research scientists at universities or large, R&D-intensive firms typically have a greater awareness, access, and ability to effectively exploit scientific knowledge originating outside of their direct research vicinity. In contrast, non-academic professionals and small-and-medium-sized enterprises (SMEs) are more likely to struggle with effective knowledge exploitation. Ware and Monkman^17^ found that half of surveyed small businesses had difficulty accessing academic research useful to their business. Houghton et al.^15^ showed that 68% of Danish firms reported access difficulties to scientific research. Galasso and Schankerman^18^ found that particularly small firms benefit from patent invalidation from large firms. Lastly, Doblinger et al.^19^ showed that small firms are more resource-constrained than larger firms and therefore benefit substantially from joint development with government partners in terms of patent production. Therefore, research shows that access, internal capabilities, and external support shape the ability of firms to exploit relevant scientific knowledge.^20^

To explore the effect of this mandate, we first build a unique dataset of more than 300,000 scientific articles from the top 20 journals (in terms of the number of DOE-funded publications) in which DOE-funded research has appeared according to the DOE’s own PAGES database. We then check for each article whether it is available without a paywall^21^ and whether and how often it got cited by patents. Finally, we use difference-in-difference(s) econometric modeling in line with the literature^22^^,^^23^ to explore the impact of the exogenous change to OA availability of NL articles on scientific research and patented inventions.

### Case selection: The DOE public access plan

**Figure 1:**
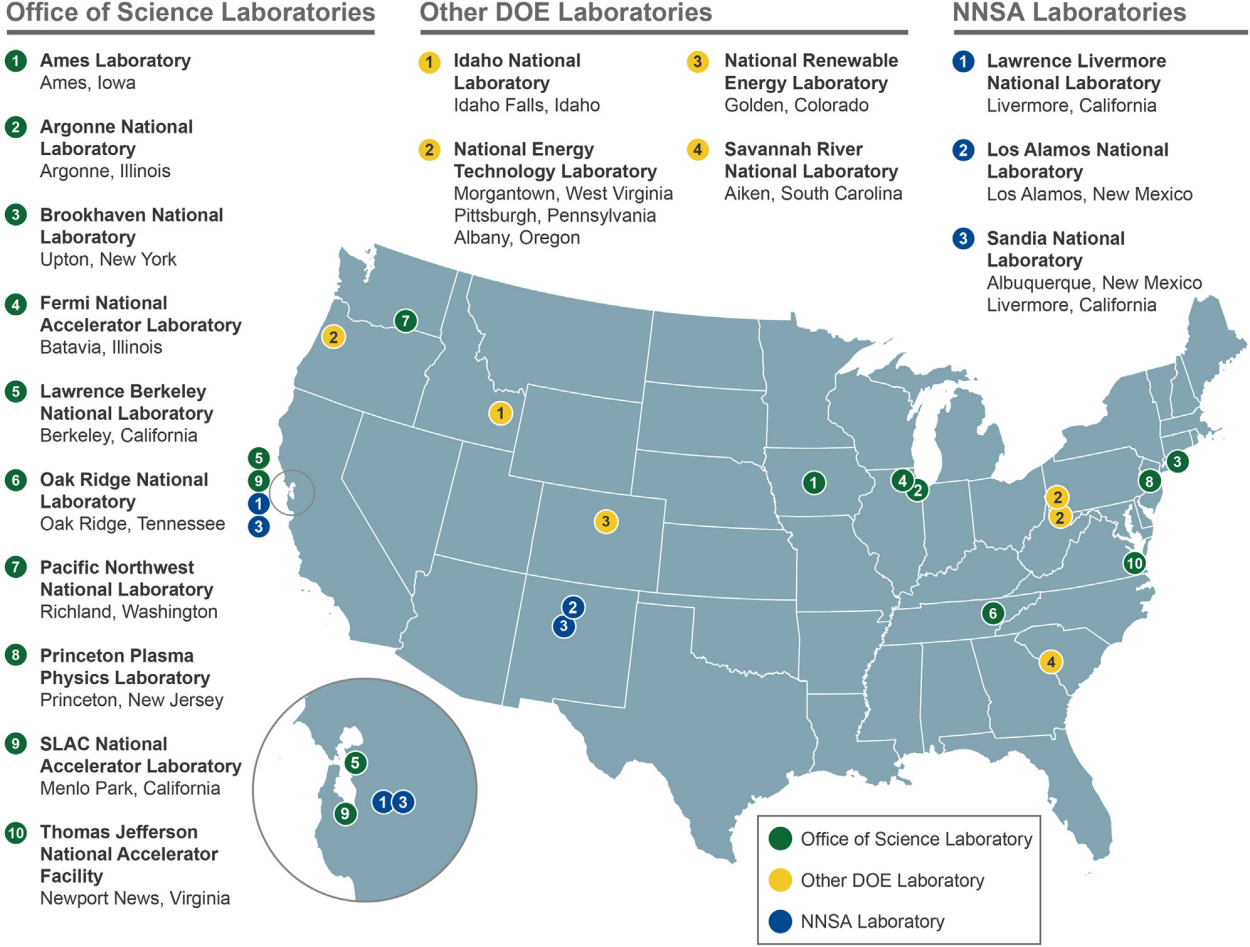
*Location and sub-groups of U.S. National Laboratories* *NNSA stands for National Nuclear Security Agency. Source:* DOE.^27^

The public sector in the U.S. plays a distinct role in advancing innovation, particularly through different ways of funding research and development (R&D) activities.^5^^,^^24^ In 2010, 31% of total R&D expenditures in the U.S. were funded by federal agencies, which has fallen to 19% in 2020.^25^ Yet, of overall government-funded R&D, one-third is conducted by federal agencies in its federally funded research centers (FFRDCs). Among the FFRCDs are 16 of the 17 NLs, commonly called “the Labs”. The NLs span the geography of the U.S. and are active in areas including nuclear weapons, fundamental research, and applied energy research (Figure 1). These received around 19 USD billion in funding in 2021^26^ and are considered the “crown jewels” of innovation and form a critical part of the energy innovation sphere of the U.S.^7^

Reviews of the NL’s performance have highlighted several areas of weak organizational design, specifically on technology transfer. Most troubling is the declining engagement with the private sector, which ultimately must adopt, develop, and deploy the energy technologies invented with public R&D. Anadon et al.^7^ document a range of declining technology transfer outcomes over the last two decades, such as NL invention disclosures and patent grants.

A potential avenue to increase the uptake of scientific advances in the private sector is to lower access costs. The DOE’s OA mandate is part of a larger plan by the U.S. federal government to increase access to federally funded science. In 2013, the White House Office of Science and Technology Policy published a memo that government-funded science should be freely accessible.^28^ In 2014, the DOE (2014) followed with its own “Public Access Plan”.^29^ In detailed guidance online, the DOE (2022; p.20^30^) explains that: “For publications emanating from DOE national laboratories and other DOE facilities, DOE requires public access to any scholarly publication published on or after October 1, 2014” and “free, public access to the full text is enabled through DOE PAGES and OSTI.GOV within 12 months after publication in a journal.”

In addition, it stated that “The OA version may be a copy of the published version of the paper if the journal permits it, a link to permanent OA version at a journal or other repository, or the author’s final peer-reviewed manuscript”.^28^ The OA policy applied to all publications from the Labs from October 2014 onwards, whereas for DOE-funded science outside the Labs, the requirements were only added to grantees for awards after October 2014. Hence, the identification is cleaner for NLs than for all grantees since there could be a substantial lag between the award of a grant and the resulting publications. It is this exogenous mandate that we exploit to address possible selection bias by researchers, who may pay for their best research to be available without a paywall.

## Results

This section provides an overview of our main results, placebo tests, and robustness checks performed in our analysis. We first discuss the changes to the publishing strategy of scientists caused by the mandate. Next, we highlight changes to the uptake of scientific knowledge by patent holders as a proxy for technological applications of the increased diffusion of science from the NLs. Lastly, we investigate who benefited primarily from the increased openness of NL studies.

### Changes in NL researchers’ publication strategy

**Figure 2:**
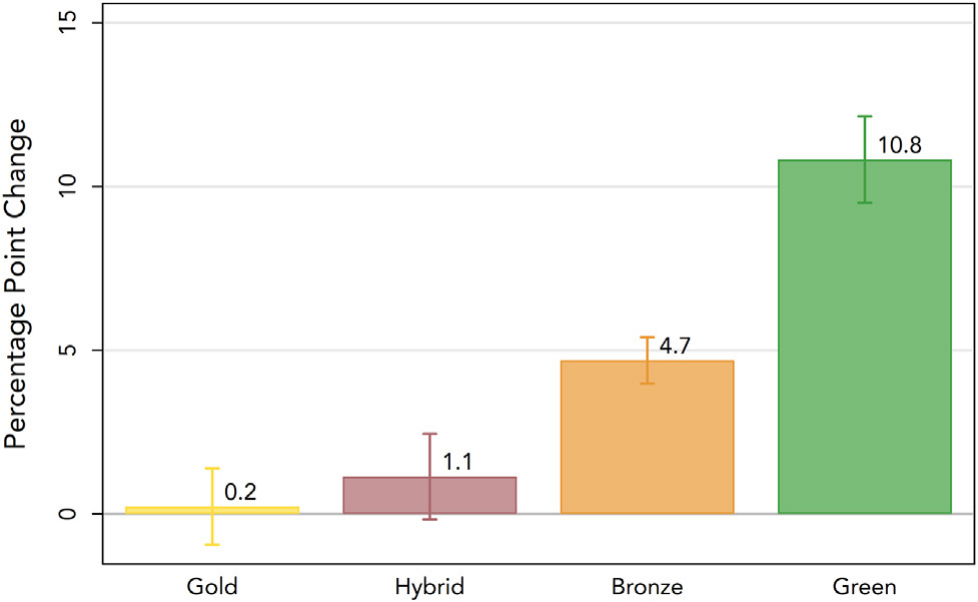
*Change in the likelihood of National Laboratory researchers publishing* via *different open-access routes after the mandate* Notes: Marginal effects estimates based on multinomial logit estimates of Equation 2 where the choice alternatives are defined as gold, hybrid, bronze, and green OA, relative to the “closed” category, which is constrained to zero. Gold OA are those journals that offer immediate access to published content, hybrid OA are open-access articles in paywalled journals for which the author paid an article processing charge. Bronze OA are articles that are free to read on the publishers’ website but without an identifiable license, which limits re-use. Lastly, green OA are paywalled articles, for which an accepted version has been deposited in a free and open repository. The full regression output is presented in Table S2. Error bars indicate 95% confidence intervals. Unit of analysis is the article level (N = 352,721).

We analyze to what extent the likelihood of different OA types changed through the OA mandate (Figure 2 see caption for a description of different OA types). If the OA mandate primarily increased green forms of OA, then this could create a substantial additional lag between the publication of the article and its availability in a free repository. We estimate a multinomial logit model of Equation 2 where the dependent variable is the categorical variable capturing the choice between bronze, green, hybrid, and gold forms of OA (with “closed” serving as the base category) to explore which OA types became more likely within the journals we analyzed (relative to our control group, which was unaffected by the mandate). The mandate primarily increased the likelihood of OA in the green category by 10.8% points, significant at the 1% level. Yet, we also find that the bronze category increased substantially (4.7% points), which are articles that are free to read on publishers’ websites but without a clearly identifiable license, which does not allow re-use of the article.^8^ In contrast, the likelihood that researchers paid for OA in the paywalled journal marginally increased by 1.1% points, significant at the 10% level. Lastly, there was no significant change in articles published in gold OA journals covered by our analysis.

### Changes in the use of National Laboratory research in scientific papers and patents

Overall, we find a strong and significant effect of the mandate on the use of NL research in patents. We differentiate between the location where the patent cites the scientific article, namely the front and body of the patent. Front citations are more likely to be added by patent lawyers, whereas body citations are more likely to be added by the inventors themselves. Body citations are typically seen as a better metric to measure knowledge diffusion as the inventor is more likely to be aware of the cited knowledge (see data section for in-depth discussion).

The number of citations received from the body of patents increased on average by 42% in the post-October 2014 period and is significant at the 1% level. Citations from the front of the patent increased but were not significant at the 5% level. In contrast, NL scientific literature was not cited more on the front of patents. Lastly, the mandate did not affect academic citations. We expected to see at least some positive effect on academic citations as indicated by a sizable body of literature investigating the “citation advantage” of OA publications.

**Figure 3:**
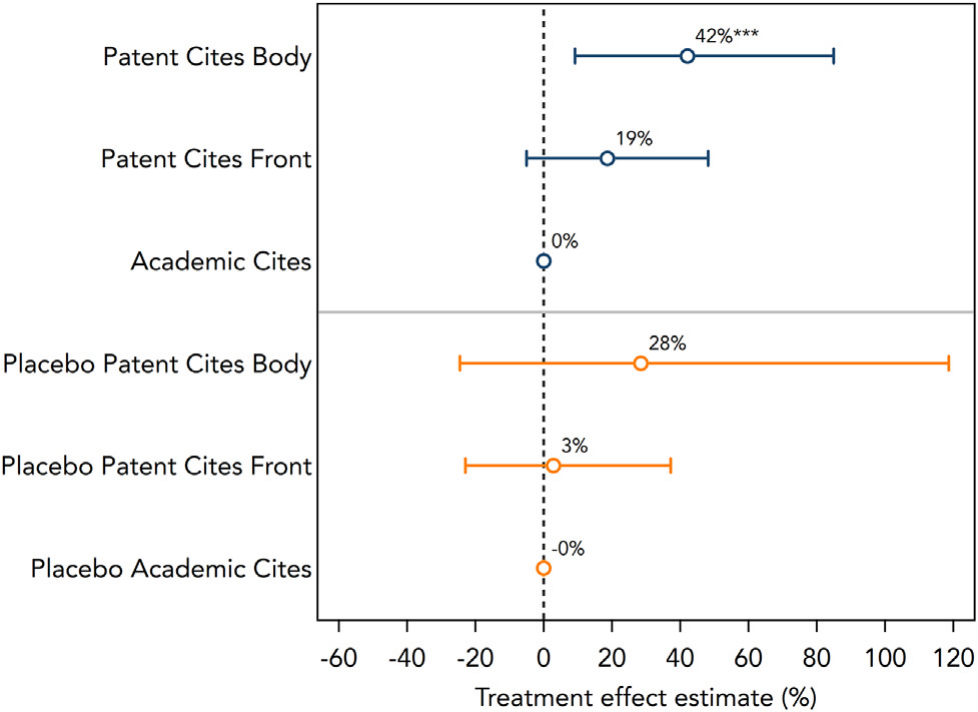
*Results visualization from main results and placebo tests* Notes: Main results and placebo analysis. The top panel presents the treatment effect estimates (in % change) for the sample of academic articles published in journals that do not routinely make their articles free to read (N = 271,753). The bottom panel presents the treatment effect estimates from our placebo analysis using the sample of academic articles published in journals that routinely make their articles OA (N = 80,968). For ease of interpretation, treatment effects are displayed in % change, based on Poisson pseudo maximum likelihood estimates of Equation 2, controlling for month-by-year fixed effects and the number of academic citations. The % change is calculated as $\left[e^{\delta^{DD}-1}\times 100\right]$. Error bars represent 95% confidence intervals. The unit of analysis is the article level (N = 352,721).

A synthesis of the main regression results and the placebo test can be found in Figure 3. Overall, we find sizable and statistically significant evidence that the OA mandate was responsible for the increased uptake of peer-reviewed academic studies in patents. While the placebo tests trend in the same direction, they are not statistically different from zero, whereas our main results are statistically significant at the 1% level. We perform a range of additional robustness checks reported in the appendix (Tables S3–S6, S10, and S11).

### Beneficiaries of increased openness of NL science

**Figure 4:**
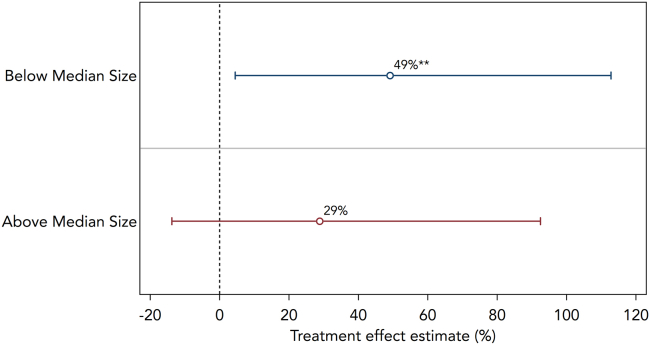
*Percentage increase in body-only patent citations by assignee size* Notes: Treatment effect estimates (in % change) for the sample of academic articles published in journals that do not routinely make their articles free-to-read (N = 271,753), split into two sub-groups corresponding to above median and below median assignee size. For ease of interpretation, treatment effects are displayed in % change, based on Poisson pseudo maximum likelihood estimates of Equation 2, controlling for month-by-year fixed effects and the number of academic citations. The % change is calculated as $\left[e^{\delta^{DD}-1}\times 100\right]$. Error bars represent 95% confidence intervals. The full regression output can be found in Table S7.

Lastly, we analyze who mainly benefited from the use of the greater openness of NL research. To proxy the firm size of the filing patent, we divide the patentees based on their patent portfolio size. As there are some patentees with large portfolio sizes, we use the median to divide the firms into “large” and “small” patentees. This approach is in line with the literature^18^ and allows us to investigate who started citing more NL research after the mandate came into force. We find strong and significant evidence that patentees with small patent portfolios cited NL research 49% more relative to a credible control group, whereas the effect on large patentees is statistically indistinguishable from zero (Figure 4).

## Discussion

Policymakers have enacted mandates that reflect the importance of free and public access to government-funded research and its role in technological innovation.^12^^,^^31^^,^^32^ Here, we investigate the impact of an OA mandate applying to the “crown jewels” of energy innovation in the U.S., namely the DOE National Labs.^7^ Our study sheds light on how an exogenous policy change to publishing rules affected the diffusion of scientific knowledge by studying the outward diffusion of scientific research from the NLs.^13^

While we focus on one specific case, we believe that it has the potential to illustrate mechanisms of knowledge diffusion that generalize beyond our context. For instance, National labs and publicly funded science play a key role across different energy innovation ecosystems.^33^ Several countries have national labs, such as the European Commission Joint Research Center (JRC), the Korea Institute of Energy Research (KIER), and the Australian Commonwealth Scientific and Industrial Research Organization, among others.

Research from other fields also supports our findings. Bryan and Ozcan^22^ study the impact of an open-access mandate in the biomedical field. They show that the National Institute of Health open access mandate led to an increase in follow-on citations from patents by 12–27%, in line with our results. Lastly, a large literature from economics and innovation research indicates that access barriers limit the diffusion of knowledge (e.g., see Keller^34^ for an excellent overview). These literature streams highlight the fundamental principles that removing barriers to access increases the diffusion of knowledge.

We make three key contributions. First, we investigate how the mandate has affected the publication strategies of NL researchers, which is important to understand as it affects the time lag with which inventors can access scientific research emanating from the Labs. Second, we explore how the change to publication rules affected other scientific research and downstream inventions, which is critical as the NLs have struggled to increase the outward diffusion of scientific research.^7^ Third, we study who benefited most from the OA mandate, shedding light on potential distributional effects. We discuss each of these contributions in turn. Lastly, we examine the limitations of our study and discuss avenues for future research.

### Changes in publication strategy

Understanding how scholars change their publishing strategies due to an OA mandate is important in several ways: First, if they opt for costly hybrid OA, this may divert research funds away from the core research to pay for publishing fees.^8^ Second, if authors opt for free, green OA, research may have lower visibility and higher access barriers due to embargo periods.^35^^,^^36^ Lastly, if they increasingly opt for gold OA, this might signal a more fundamental shift in the publishing strategy of scholars, indicating a move toward full models of OA.

Our results indicate that NL researchers have primarily responded to the mandate by self-archiving their articles (green OA), which has important implications for our results and the impact of the policy itself. Hybrid and gold OA allow for immediate, free, and public access to research articles, yet depositing accepted articles in repositories (green OA) commonly comes with the requirement of an embargo period of several months to over one year.^35^^,^^36^ Publishers claim that the embargo period is critical, otherwise, libraries and research institutions might decide to cancel their journal subscriptions, leading to a substantial loss of revenue.^8^ Researchers subject to the OA mandate must make the article free to read within one year of publication, but many journals preclude authors to deposit accepted manuscripts in repositories upon publication in a journal.

Our estimates of the NL mandate’s impact on downstream inventions should therefore be seen as the likely lower bound as impacts would likely even be higher if articles were free to read upon publication. We also observe imperfect compliance with the mandate, suggesting that our estimated impacts could even be larger in the case of full compliance (around 10% of NL authors do not comply with the mandate in our study period).

Lastly, we also document an increase in Bronze OA, which are articles that have no clearly identifiable license but are free to read on the publishers’ website. While these articles can be accessed without cost, the lack of a clear license means that they cannot be re-used.^8^ In addition, for Bronze OA the rights remain with the publishers, making it unclear whether OA is granted temporarily or permanently, which could lead to unstable diffusion through this OA pathway.^4^^,^^37^

Our results hold important implications for policymakers. First, the exact policy design significantly influences the diffusion of scientific knowledge. Given that scholars strive to minimize the cost of complying with the mandate, the imposed embargo periods further delay public access to scientific publications. Therefore, the U.S. government’s decision to require immediate public access to federally funded publications from 2026 onwards is a critical step toward ensuring timely access.^12^ Second, compliance enforcement is another key component in increasing the diffusion of scientific knowledge. We found that approximately 10% of researchers do not comply with the mandate. Thus, policymakers should prioritize clear communication of the requirements and consider implementing penalties for non-compliance. Lastly, the rise in articles published under a bronze license—which are free to read but lack a clearly identifiable license—signals a need for policymakers to collaborate more closely with publishers to ensure clear licensing of scientific articles.

### Outward diffusion of NL scientific knowledge into academic research and patented inventions

Scientific knowledge entails both tacit and explicit knowledge and various diffusion channels, which makes it difficult to investigate. In our study, we leverage a range of recent advances in data availability by Marx and Fuegi^38^^,^^39^ which standardize and report the connections between patents and scientific publications on a large scale. Combining bibliometric, patent, and open-access data on a large scale, we demonstrate the causal effect of the policy on academic research and downstream inventions.

Our results indicate that the OA mandate is most useful in spurring additional technical inventions but not additional scientific research (as proxied by additional citations from research articles). Our results suggest that at least on average researchers do not struggle to access scientific research, though there might be inequities that our results do not fully reflect (e.g., researchers from low-income countries and other potentially disadvantaged groups). The application of NL research in patented inventions is particularly important for the NLs as they have historically struggled with increasing the outflow of knowledge to more applied domains.^7^ While there is a range of important diffusion channels apart from scientific publications,^40^ increasing the diffusion of scientific knowledge—via scientific papers—through open-access mandates appears to be an effective strategy to increase outward diffusion.

### Beneficiaries of the increased scientific knowledge diffusion

Lastly, we find that particularly small firms (those with below-median patent portfolios) benefited from the OA mandate. After the mandate, small firms cite NL research around 50% more (compared to articles that were not subject to the mandate). This change indicates that particularly smaller firms are adversely affected by the journals’ paywalls. Several survey-based studies indicate access barriers, but we are the first study to demonstrate the effect of an OA mandate on downstream patented inventions by smaller firms. We, therefore, make an important extension to the work of Galasso and Schankerman (2015) who show that patent invalidation particularly helps small firms. Yet, we document that this also holds for the diffusion of scientific knowledge, which tends to be further removed from the actual application than the patent-to-patent citations that Galasso and Schankerman (2015) draw on for their analysis.

SMEs play an integral role in driving economic development in Europe and the U.S. but appear to be particularly affected by difficult access to scientific publications.^15^ In the U.S. SMEs contribute 44% of U.S. economic activity, though their share has declined over time.^41^ Similarly, Europe’s economy is underpinned by 25 million SMEs employing around 100 million people and contributing more than half of Europe’s GDP.^42^ While an OA mandate may not seem to be an SME promotion policy, it is important to stress that these appear to be the key beneficiaries, though more work needs to be done to explore the exact mechanisms behind the increased uptake and the use of patents in this subgroup.^20^^,^^43^ While we do not show this empirically, high-growth start-ups are likely not the main beneficiaries, as they are often spun out from universities,^44^ which should give them easier access to academic publications. In contrast, SMEs are likely further removed from the academy.^15^

### Future research

Our work can be extended in several ways. First, it would be critical to investigate more closely how OA mandates impact other forms of knowledge diffusion.^13^ While the OA mandate primarily affected the ways peer-reviewed scientific publications are stored and used, the mandate may have led to the additional transfer of tacit knowledge due to subsequent academic consulting, co-supervision of students, or other science-industry spillovers.^45^ Understanding these complementary knowledge transfers is critical^40^ but difficult to study. Similarly, while paywalls typically only cover the peer-reviewed article, the DOE also encourages researchers to deposit their data for further use. Analyzing to what extent data in repositories is re-used and sparks novel research and technological applications, may be another fruitful avenue to pursue.^20^

While we show that the mandate primarily affected patented inventions by small firms, studying whether academics from low- and middle-income countries benefited from the mandate, leading to spillovers outside of the U.S. would be an important addition to the literature.^34^ Understanding where and how the beneficiaries are located, how they use the knowledge, and what economic consequences it has, is important to understand. Estimating the increased return to R&D due to greater knowledge spillovers would be another interesting research avenue.^8^ Lastly, investigating whether the patents were eventually commercialized would be a further step toward understanding the full effect of the policy. Recent advances in virtual patent marking papers could be a promising research avenue.^46^

### Limitations of study

Our work draws on one diffusion channel of scientific research, namely, scientific publications. Yet, there are many ways that scientific knowledge can diffuse, including co-supervision of students between industry and academia, licensing, and consulting.^13^^,^^14^^,^^16^ Hence, our work covers only one potential pathway for diffusion, yet one that is commonly seen among the most important diffusion channels.^15^ In addition, as is commonly challenging in innovation research, we can only follow the “paper trail”, meaning that patents offer a standardized, well-documented data source for studying the diffusion of scientific knowledge into more practical applications. Yet, while patents are widely used in the innovation literature, they come with certain limitations.^47^ Not all inventions are patented, and some firms may choose to protect their inventions by secrecy and lead times, rather than by patents.^13^ While our results indicate that smaller firms benefited most strongly from the NL mandate, more work in this regard is needed, particularly in understanding how small firms find, understand, and use knowledge emanating from research facilities.^20^

## STAR★Methods

### Key resources table

**Table:** 

REAGENT or RESOURCE	SOURCE	IDENTIFIER
**Software and algorithms**		

Stata 17	Stata	https://www.stata.com/stata-news/news36-2/
DRDID	Rios Avilla et al. 2022	https://friosavila.github.io/playingwithstata/main_drdid.html

### Resource availability

#### Lead contact

Further information requests should be directed to the lead contact Benedict Probst (bprobst@ethz.ch).

#### Materials availability

This study did not use or generate reagents.

### Method details

#### Data

The data compilation process is shown in Figure S3 and contains five steps. We first compiled a list of the top 20 journals that DOE-funded research has appeared in the past according to the PAGES database (a full list can be found in Table S8). In a second step, we downloaded all articles from the Dimensions.AI database that appeared in these journals between 2012 and 2018, which gives us at least three years before and after the mandate (352,721 articles). For each of the scientific articles, we check whether at least one author is associated with one of the Labs and therefore was subject to the OA mandate. Third, we used the 'reliance on science’ database to check whether articles are cited by a patent or not.^38^^,^^39^ The database differentiates between patent citations to academic articles that stem from the body or the front of the patent. Citations from the body of the patent are considered to be a better proxy for knowledge diffusion as these citations are commonly added by inventors rather than patent lawyers. Fourth, we used the Unpaywall (2019)^21^ API to check for each article (via its digital object identifier) whether the article is available without a paywall. Unpaywall is a comprehensive database for the OA status and is widely used in the literature (Else, 2018b; Unpaywall, 2019). Fifth, we scraped Google Patents to determine the size of the patent assignees’ portfolio (of patents that cite scientific research). Our final dataset contains 352,721 scientific articles published between 2012 and 2018. The descriptive statistics of the dataset can be found in Table S9.

Inventors can cite scientific literature in two parts of the patent, namely the ‘front’ of the patent and the ‘body’ of the patent. The latter is also called in-text citations in the literature. Several studies^22^^,^^48^ indicate that citations from the body of the patent may better reflect true knowledge flows between scientific research and patented technologies as these citations are more commonly added by inventors rather than patent examiners. The inventor may be unaware of citations to the academic literature added by patent examiners and that citation therefore would not constitute the true state of the inventors’ knowledge when filing the patent. Bryan and Ozcan (2021) therefore use body citations to track the diffusion between peer-reviewed scientific articles and patented inventions but do not test whether citations from the front of the patents also become more likely because of OA mandates. To test for differences in the effect of these different types of citations, we include both in our empirical analysis.

### Quantification and statistical analysis

To estimate the causal effect of the NL mandate on patent citations we use a difference-in-differences (DID) model for two main reasons. First, to address selection bias, which is a key concern in causal inference. In our case, researchers could pay to make their best articles published open access. This self-selection can confound the estimates of the mandate’s effect. A DID-model controls for time-invariant confounders that simultaneously influence the selection into open access and the outcome. Second, to tackle time-varying confounders. Before and after the implementation of the policy, there might be concurrent changes in the wider political, technical, and funding landscape. By comparing changes in the treatment group (subject to the mandate) to a credible control group (not subject to the mandate, but otherwise affected by the same broader changes), the DID model can isolate the causal effect of the policy by addressing these time-varying confounders.

As a baseline specification, we estimate a canonical DID model with a binary treatment indicator and a binary post-treatment indicator. The unit of analysis is at the individual article level, denoted by the subscript i*:*

(Equation 1) $$Y_{i}=\rho NL+\gamma PostOct14+\delta^{DD}\left(NL\times PostOct14\right)+\varepsilon_{i}$$

where $Y_{i}$ is the dependent variable capturing the number of article-level patent citations (emanating from the body or front of the patent), or the number of academic citations. NL is the treatment indicator identifying articles that were published by authors affiliated with the National Labs. PostOct14 is the post-treatment indicator identifying articles that were published on or after 1^st^ October 2014, the date from which NL articles had to be published OA. The DID estimator ($\delta^{DD}$) is obtained from the interaction of both main effects (NL×PostOct14) and provides an unbiased estimate of the effect of the NL OA mandate on the dependent variables (Note that the DID estimator captures the effect of the NL OA mandate on patent citations, not the effect of OA per-se. The estimate thus reflects the incremental effect of increasing the share of OA articles published by NLs by 30 percentage points (from 60% to 90%) compared to the control group that saw little change in the share of OA articles.).

To probe the robustness of our baseline specification, we estimate a generalized DID model, which additionally captures general trends in patent citations over time using a set of temporal fixed effects. Moreover, we include relevant article-level control variables:

(Equation 2) $$Y_{i}=\rho NL+\delta^{DD}\left(NL\times PostOct14\right)+\lambda_{t}+X_{i}+\varepsilon_{i}$$

where $Y_{i}$ is the dependent variable capturing the article-level citations (body, front and academic) (The article-level citations refers to whether the citation to the scientific article emanates from the body of the patent, the body of the patent, or a scientific article (see data section for detailed description).). For our analysis of OA type (Hypothesis 1), $Y_{i}$ is a categorical outcome variable that captures four OA publishing alternatives (hybrid, green, bronze and gold) relative to ‘closed’ articles (the base category). $\lambda_{t}$ are month-by-year fixed effects which account for temporal trends common to both NL and non-NL articles, and $X_{i}$ represents a vector of article-level control variables including the number of authors and the number of academic citations (inverse hyperbolic sine transformed, which is a common alternative to log-transformation in the presence of 0-values), a measure of article quality, as well as the journal in which the article was published (using a set of journal dummies). The journal fixed effects capture all journal-specific unobserved characteristics (e.g., Journal quality, readership and reach).

We estimate Equations 1 and 2 using Poisson Pseudo Maximum Likelihood under the assumption that the OA mandate caused a multiplicative increase in total patent citations (i.e., following a 0-inflated Poisson distribution). Importantly, the generalized DID model provides unbiased estimates of the treatment effect in settings where there is a single treatment period (i.e., no staggered implementation of the treatment) (See Baker et al. (2022)^49^ for details.). DID models are sensitive to the parallel trends assumption. In support of parallel trends, we find no significant pre-mandate differences in the number of body patent citations between treatment and control groups, aggregated over six-month periods (see Figure S1). Moreover, we use the approach developed in Rambachan and Roth (2023)^50^ to show that our results are robust to violations in the parallel trends assumption of up to 5% (see Figure S2). Finally, we use the doubly robust difference-in-differences estimator (DRDID)^51^ to test the robustness of our main results. The doubly robust estimator combines both a regression adjustment approach and an augmented inverse-probability weighting approach, which produces consistent and robust estimates even if either the outcome regression model or the propensity score model are misspecified.^51^

A key hypothesis underpinning our study is that the OA mandate effectively increased the OA likelihood of studies emanating from NLs but not from studies coming from non-NLs. As Figure S4 shows, the OA likelihood of studies between NL and non-NL research groups was very similar before the mandate, oscillating between 50% and 60%. After the introduction of the mandate, the OA likelihood increased to around 90% for NLs, whereas non-NLs saw only slight increases relative to the baseline of around 60%. Overall, the mandate increased the OA likelihood relative to the baseline by 30%-points. As our study investigates only this marginal change in the OA availability (and not the difference between fully closed vs. fully open), the true empirical effect of OA articles could be up to three times as large (if one assumes linear scaling of effects).

All models are estimated using Stata 17. Full regression outputs can be found in the Appendix, which accompany the visualizations presented in the manuscript. We estimate the DRDID using the user-written Stata package ‘drdid’ (Rios-Avila et al., 2022). Results are presented in Table S11. Please note that the outcome regression model is estimated by ordinary least squares (OLS) and coefficient estimates thus differ in magnitude from our main analysis outcomes obtained from Poisson Pseudo Maximum Likelihood estimation. In the figures, error bars indicate the 95% confidence intervals. Significance levels indicated by ∗∗∗ (1%), ∗∗ (5%) and ∗ (10%).

## Data Availability

• The data are available upon request from the lead author. • The code is available upon request from the lead author. • Any additional information required to reanalyze the data reported in this paper is available from the lead contact upon request.
