# Supplementary material for: The impact of open access mandates on scientific research and technological development in the U.S
Source: iScience. 2023 Aug 26;26(10):107740. doi: 10.1016/j.isci.2023.107740 (PMC10504483; doi:10.1016/j.isci.2023.107740)
Supplement: Document S1. Figures S1–S4 and Tables S1–S11 [file mmc1.pdf]

iScience, Volume *26*

Supplemental information

The impact of open access mandates on scientific research and technological development in the U.S.

Benedict Probst, Paul M. Lohmann, Andreas Kontoleon, and Laura Díaz Anadón

Supplemental Figures

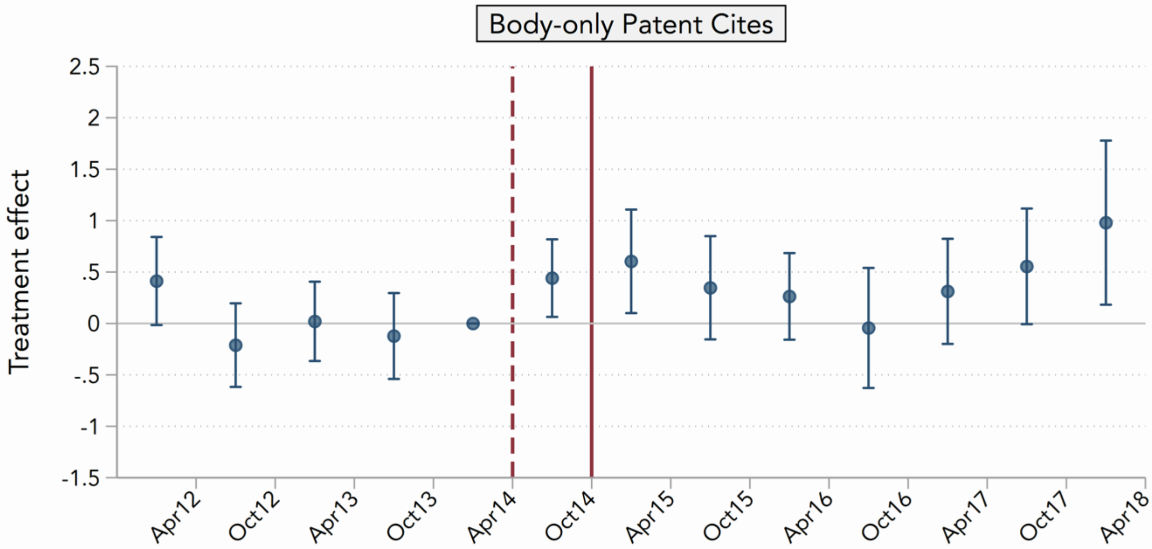

Figure S1: Event study estimates, related to Figure 3.
Notes: Figure plots the difference in the number of patent citations between NL and non-NL articles across all six-month intervals before and after the NL Open Access Mandate (introduced in October 2014). The six-month interval directly prior to the mandate reflects the anticipation period, following the announcement of the mandate. Point estimates are raw Poisson Pseudo Maximum Likelihood estimates. Article level controls include the number of authors, the number of academic citations and journal fixed effects. Error bars represent 90% confidence intervals.

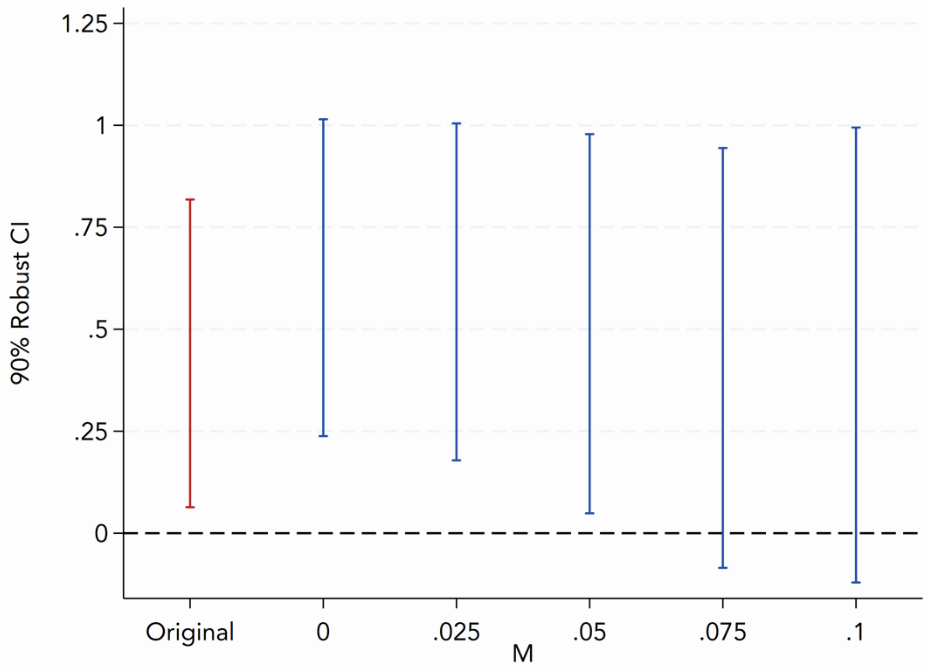

Figure S2: Violations to the parallel trends assumption and our main result estimates, related to Figure 3. Implemented with the user-written ‘honestdid’ Stata command. Source: authors.

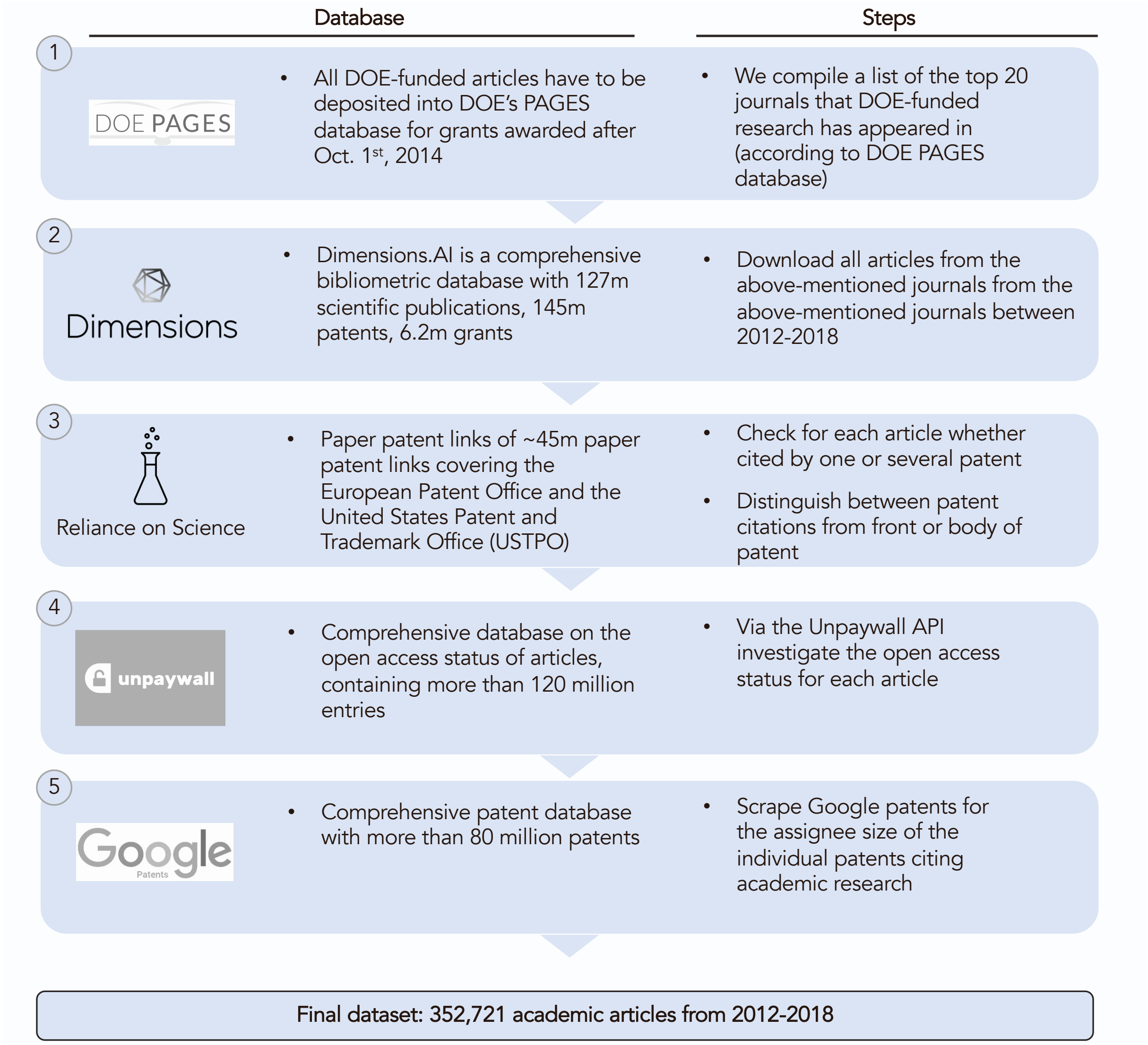

Figure S3: Steps and key databases involved in creating the dataset used in our research, related to Figure 3.

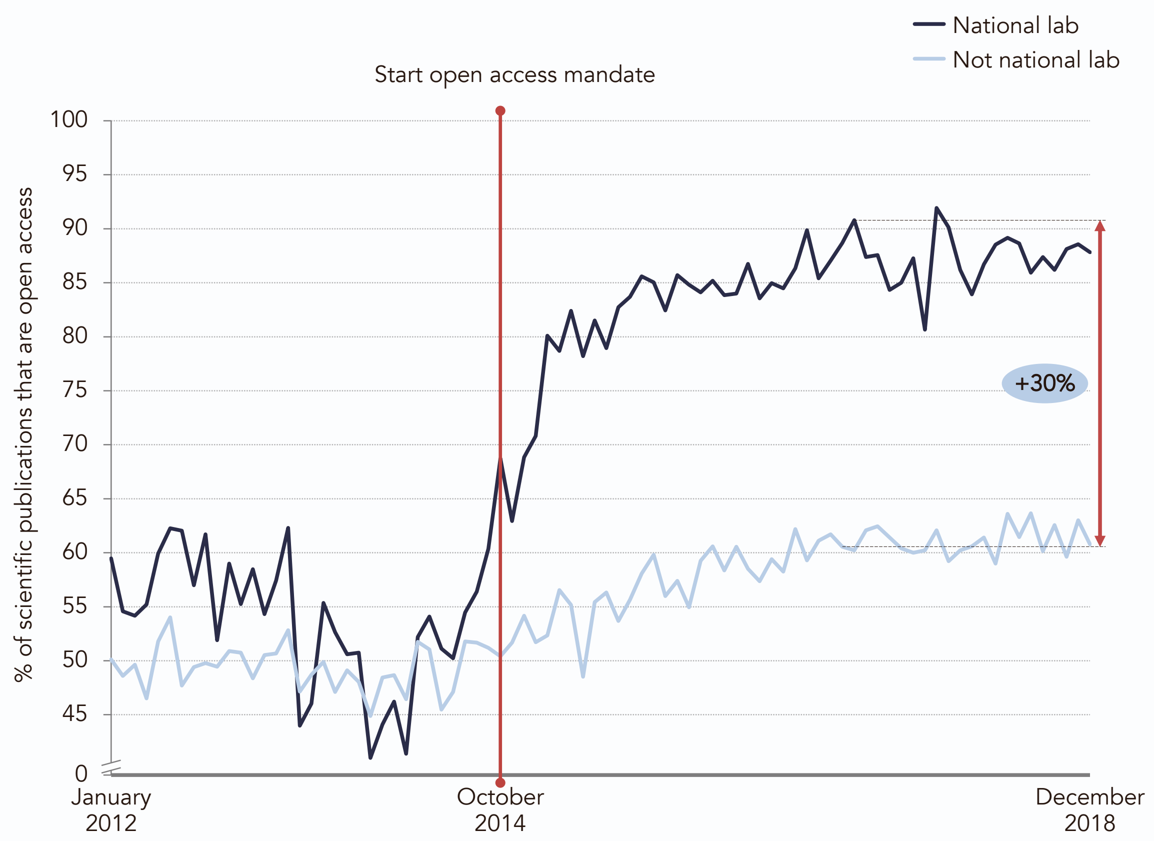

Figure S4: Percentage of open-access publications emanating from National Laboratories and non-National Laboratories, related to Figure 3. *Figure shows the share of OA in the 16 paywalled journals of our dataset (excluding 4 open-access journals, which were not 'treated' by the mandate).*

Supplemental Tables

Table S1: OA types in pre-mandate period, related to Figure 2.

	Control	Treated	Difference	N
Closed	0.438	0.426	-0.012**	134,162
Hybrid	0.069	0.201	0.132***	134,162
Green	0.367	0.304	-0.063***	134,162
Bronze	0.062	0.041	-0.021***	134,162
Gold	0.064	0.027	-0.037***	134,162
Observations	125,258	8,904		

Notes: Table displays the share of articles published closed or OA, disaggregated by OA type. The third column displays the difference in means and significance stars correspond to p-values obtained from a two-sided t-test for comparison of Treated and Control group means. *N* refers to the number of observations used to compute the means and conduct the t-test.

* p < 0.10, ** p < 0.05, *** p < 0.01

Table S2: Multinomial Logit Estimates – OA Types, related to Figure 2.

	(1) Gold	(2) Hybrid	(1) Bronze	(2) Green
National labs × Post Oct. 2014	.0022	.0114*	0.047***	0.108***
	(0.005)	(0.007)	(0.004)	(0.007)
Month-Year FE	Yes	Yes	Yes	Yes
N	352,721	352,721	352,721	352,721

Notes: Marginal effects based on multinomial logit estimates of equation (2), excluding controls. The base category is the 'closed' category, which is constrained to zero. Robust standard errors in parentheses.

* *p* < 0.10, ** *p* < 0.05, *** *p* < 0.01

Table S3: Robustness – First author origin co-variate, related to Figure 3.

	(1) Body-only Patent Citations	(2) Front-only Patent Citations	(3) Academic Cites
National labs × Post Oct. 2014	0.318**	0.149	0.007
	(0.134)	(0.113)	(0.004)
In % change	37	16	1
National labs	-0.427***	-0.370***	0.040***
	(0.071)	(0.066)	(0.004)
Month-Year FE	Yes	Yes	Yes
Controls	Yes	Yes	Yes
Observations	271,753	271,753	271,753

Notes: Poisson Pseudo Maximum Likelihood estimates of equation 2. The sample consists of articles published in sixteen journals that do not routinely make their articles free to read. *National labs* is the treatment indicator identifying articles published by authors affiliated with the National Labs, and *Post Oct. 2014* is the post-treatment indicator, identifying articles published after October 2014. The interaction *National labs × Post Oct. 2014* produces the treatment effect of the NL mandate on citations. Article level controls include the first author country of origin, the number of authors, the number of academic citations and journal fixed effects. First author countries with few observations (N<500) or missing information (N=8310) were recoded as 'other', to limit the number of dummies included in the model and retain statistical power. The effect in % change is calculated as [eDD-1 ×100]. Robust standard errors in parentheses.

* *p* < 0.10, ** *p* < 0.05, *** *p* < 0.01

Table S4: Robustness – Excluding Non-NL DOE-funded articles (N=15,251), related to Figure 3.

	Body-only Patent Citations		Front-only Patent Citations		Academic Cites	
	(1)	(2)	(3)	(4)	(5)	(6)
National labs × Post Oct. 2014	0.393***	0.371***	0.210*	0.173	-0.003	0.005
	(0.142)	(0.135)	(0.119)	(0.114)	(0.005)	(0.004)
In % change	48	45	23	19	-0	1
National labs	-0.288***	-0.176**	-0.183***	-0.178***	0.054***	0.071***
	(0.076)	(0.070)	(0.069)	(0.065)	(0.004)	(0.003)
Post Oct. 2014	-1.684***		-1.113***		-0.100***	
	(0.047)		(0.037)		(0.002)	
Month-Year FE	No	Yes	No	Yes	No	Yes
Controls	No	Yes	No	Yes	No	Yes
Observations	256,977	256,977	256,977	256,977	256,977	256,977

Notes: Poisson Pseudo Maximum Likelihood estimates of equation 1 (columns 1, 3 and 5) and equation 2 (columns 2, 4 and 6). The sample consists of articles published in sixteen journals that do not routinely make their articles free to read and excludes DOE-funded articles which were published by non-NL authors. *National labs* is the treatment indicator identifying articles published by authors affiliated with the National Labs, and *Post Oct. 2014* is the post-treatment indicator, identifying articles published after October 2014. The interaction *National labs × Post Oct. 2014* produces the treatment effect of the NL mandate on citations. Article level controls include the number of authors, the number of academic citations and journal fixed effects. The effect in % change is calculated as [eDD-1 ×100]. Robust standard errors in parentheses.
* $p < 0.10$, ** $p < 0.05$, *** $p < 0.01$

Table S5: Robustness – 3-year pre-post interval, related to Figure 3.

	Body-only Patent Citations		Front-only Patent Citations		Academic Cites	
	(1)	(2)	(3)	(4)	(5)	(6)
National labs × Post Oct. 2014	0.349**	0.316**	0.180	0.128	0.002	0.003
	(0.147)	(0.140)	(0.126)	(0.121)	(0.005)	(0.005)
In % change	42	37	20	14	0	0
National labs	-0.239***	-0.141*	-0.121	-0.113	0.049***	0.067***
	(0.084)	(0.077)	(0.077)	(0.074)	(0.004)	(0.003)
Post Oct. 2014	-1.519***		-0.985***		-0.082***	
	(0.044)		(0.035)		(0.002)	
Month-Year FE	No	Yes	No	Yes	No	Yes
Controls	No	Yes	No	Yes	No	Yes
Observations	256,046	256,046	256,046	256,046	256,046	256,046

Notes: Poisson Pseudo Maximum Likelihood estimates of equation 1 (columns 1, 3 and 5) and equation 2 (columns 2, 4 and 6). The sample consists of articles published in sixteen journals that do not routinely make their articles free-to-read and is limited to the three-year interval before and after the mandate came into force. *National labs* is the treatment indicator identifying articles published by authors affiliated with the National Labs, and *Post Oct. 2014* is the post-treatment indicator, identifying articles published after October 2014. The interaction *National labs × Post Oct. 2014* produces the treatment effect of the NL mandate on citations. Article level controls include the number of authors, the number of academic citations and journal fixed effects. The effect in % change is calculated as [eDD-1 ×100]. Robust standard errors in parentheses.
* $p < 0.10$, ** $p < 0.05$, *** $p < 0.01$

Table S6: Robustness – Anticipatory Period: Post from July 2014, related to Figure 3.

	Body-only Patent Citations		Front-only Patent Citations		Academic Cites	
	(1)	(2)	(3)	(4)	(5)	(6)
National labs × Post Jul. 2014	0.453***	0.406***	0.219*	0.163	-0.004	0.002
	(0.132)	(0.125)	(0.115)	(0.109)	(0.005)	(0.004)
In % change	57	50	24	18	-0	0
National labs	-0.315***	-0.209***	-0.181**	-0.175***	0.051***	0.068***
	(0.079)	(0.073)	(0.073)	(0.068)	(0.004)	(0.004)
Post Jul. 2014	-1.610***		-1.091***		-0.098***	
	(0.044)		(0.035)		(0.002)	
Month-Year FE	No	Yes	No	Yes	No	Yes
Controls	No	Yes	No	Yes	No	Yes
Observations	271,753	271,753	271,753	271,753	271,753	271,753

Notes: Poisson Pseudo Maximum Likelihood estimates of equation 1 (columns 1, 3 and 5) and equation 2 (columns 2, 4 and 6). The sample consists of articles published in sixteen journals that do not routinely make their articles free-to-read. *National labs* is the treatment indicator identifying articles published by authors affiliated with the National Labs, and *Post Jul. 2014* is the post-treatment indicator, identifying articles published on or after 1st July 2014, the date on which the NL OA strategy was announced. The interaction *National labs × Post Jul. 2014* produces the treatment effect of the NL mandate on citations. Article level controls include the number of authors, the number of academic citations and journal fixed effects. The effect in % change is calculated as [eDD-1 ×100]. Robust standard errors in parentheses.
* $p < 0.10$, ** $p < 0.05$, *** $p < 0.01$

Table S7: Percentage increase in body-only patent citations by assignee size (full regression output), related to Figure 3.

	Above Median	Below Median
	(1)	(2)
National labs × Post Oct. 2014	0.400**	0.253
	(0.181)	(0.205)
In % change	49	29
National labs	-0.162*	-0.208*
	(0.093)	(0.106)
Month-Year FE	Yes	Yes
Controls	Yes	Yes
Observations	259,714	260,802

Notes: Poisson Pseudo Maximum Likelihood estimates of equation 2. The sample in column (1) consists of articles published in non-OA journals and cited by patents with above median assignee size and all uncited articles. The sample in column (2) consists of articles published in non-OA journals and cited by patents with below median assignee size and all uncited articles. *National labs* is the treatment indicator identifying articles published by authors affiliated with the National Labs, and *Post Oct. 2014* is the post-treatment indicator, identifying articles published after October 2014. The interaction *National labs × Post Oct. 2014* produces the treatment effect of the NL mandate on citations. Controls include the log-transformed number of academic citations at the article-level. The effect in % change is calculated as $[e^{\delta DD-1} \times 100]$. Robust standard errors in parentheses.
* $p < 0.10$, ** $p < 0.05$, *** $p < 0.01$

Table S8: Overview of journals in our sample, related to Figure 5. We consider a journal OA if the average OA availability of the journal was above 90% before the mandate.

**Journal Name**	**Number of publications**	**OA**
SCIENTIFIC REPORTS	60015	Yes

PHYSICAL REVIEW B (no embargo period)[1]	48297	No
PROCEEDINGS OF THE NATIONAL ACADEMY OF SCIENCES OF THE UNITED STATES OF AMERICA (embargo: 6 months for accepted/published version in institutional repository)[2]	37053	No
APPLIED PHYSICS LETTERS (embargo: 12 months for published version, 0 for accepted version)[3]	33937	No
PHYSICAL REVIEW D (no embargo period)[4]	30008	No
JOURNAL OF APPLIED PHYSICS (embargo period: 12 months for published version, 0 for accepted version)	29690	No
THE JOURNAL OF PHYSICAL CHEMISTRY C (embargo period: 12 months for accepted version)	29634	No
NATURE COMMUNICATIONS	28522	Yes
PHYSICAL REVIEW LETTERS (no embargo period)[5]	26098	No
JOURNAL OF THE AMERICAN CHEMICAL SOCIETY (no embargo period)	25008	No
THE JOURNAL OF CHEMICAL PHYSICS (embargo period: 12 months)	22124	No
JOURNAL OF HIGH ENERGY PHYSICS	17660	Yes
PHYSICS OF PLASMAS (embargo period: published version 12 months, accepted 0 months)	11019	No
REVIEW OF SCIENTIFIC INSTRUMENTS (embargo period: published version 12 months, accepted 0 months)[6]	10518	No
NUCLEAR INSTRUMENTS AND METHODS IN PHYSICS RESEARCH SECTION A ACCELERATORS SPECTROMETERS DETECTORS AND ASSOCIATED EQUIPMENT (embargo period: 24 months)	10486	No
NANO LETTERS (embargo period: 12 months for accepted and published version)	10167	No
PHYSICAL REVIEW C (no embargo period)	9686	No
CHEMISTRY OF MATERIALS (embargo period: 12 months for accepted and published version)	8086	No
PHYSICS LETTERS B	7204	Yes
JOURNAL OF NUCLEAR MATERIALS (embargo period: 24 months)	6833	No

Table S9: Descriptive Statistics (full dataset), related to Figure 3.

National Labs (Treated)	Mean	SD	Min	Max	N
OA	.75	.44	0.00	1	22,063
Pr(>=1 patent citations) (body)	.03	.17	0.00	1	22,063
Pr(>=1 patent citations) (front)	.09	.28	0.00	1	22,063
Mean # of patent citations (body)	.05	.45	0.00	25	22,063
Mean # of patent citations (front)	.28	1.93	0.00	103	22,063
Mean # of academic cites	45.3	90.38	0.00	3,517	22,063
# of authors	9.98	39.26	1.00	3,048	22,063

Non-National Labs (Control)	Mean	SD	Min	Max	N
OA	.65	.48	0.00	1	330,658
Pr(>=1 patent citations) (body)	.03	.17	0.00	1	330,658
Pr(>=1 patent citations) (front)	.09	.29	0.00	1	330,658
Mean # of patent citations (body)	.06	.7	0.00	105	330,658

[1] https://v2.sherpa.ac.uk/id/publication/13635
[2] https://v2.sherpa.ac.uk/id/publication/10338
[3] https://v2.sherpa.ac.uk/id/publication/9864
[4] https://v2.sherpa.ac.uk/id/publication/32263
[5] https://v2.sherpa.ac.uk/id/publication/13640
[6] https://v2.sherpa.ac.uk/id/publication/9874

Mean # of patent citations (front)	.31	2.57	0.00	414	330,658
Mean # of academic cites	38.64	72.95	0.00	5,803	330,658
# of authors	5.84	7.17	1.00	698	330,658

Notes: Table displays descriptive statistics for Non-National Laboratory and National-Laboratory articles published in any of the 20 journals in our dataset for the full observation period. Pr refers to probability. Front and body refer to the location of the patent from where the scientific citation was extracted. Academic cites are those received from other scientific publications.

We run a placebo test to investigate to what extent non-treated journals also exhibit similar trends in the number of increased citations. These journals had OA rates of more than 95% across the entire study period and therefore should not have been affected by the mandate. If these journals also exhibit increased citation likelihood, then the increased diffusion of scientific knowledge into patented technologies was more likely driven by NL-wide factors unrelated to the OA mandate. These could be, for example, a trend of increased collaboration with the private sector that was unrelated to the OA mandate. Table A10 shows our model for the non-treated journals, which indicates that our results are not explained by other factors that were concurrent with the OA mandate. Specification (5) shows significant effects on academic citations, but these effects disappear once month-year fixed effects and article-level controls are introduced into the model.

Table S10: Placebo tests, related to Figure 3.

	Body-only Patent Citations		Front-only Patent Citations		Academic Cites	
	(1)	(2)	(3)	(4)	(5)	(6)
National labs × Post Oct. 2014	0.175	0.250	-0.125	0.028	-0.030**	-0.017
	(0.283)	(0.271)	(0.178)	(0.147)	(0.012)	(0.011)
In % change	19	28	-12	3	-3	-2
National labs	0.260	-0.253	0.191	-0.280**	0.123***	0.078***
	(0.238)	(0.235)	(0.145)	(0.116)	(0.010)	(0.009)
Post Oct. 2014	-1.370***		-0.866***		-0.064***	
	(0.069)		(0.048)		(0.003)	
Month-Year FE	No	Yes	No	Yes	No	Yes
Controls	No	Yes	No	Yes	No	Yes
Observations	80,968	80,968	80,968	80,968	80,968	80,968

Notes: Poisson Pseudo Maximum Likelihood estimates of equation 1 (columns 1, 3 and 5) and equation 2 (columns 2, 4 and 6). The sample consists of articles published in four journals that routinely make their articles free to read. *National labs* is the treatment indicator identifying articles published by authors affiliated with the National Labs, and *Post Oct. 2014* is the post-treatment indicator, identifying articles published after October 2014. The interaction *National labs × Post Oct. 2014* produces the treatment effect of the NL mandate on citations. Article level controls include the number of authors, the number of academic citations and journal fixed effects. The effect in % change is calculated as $[e^{\delta^{DD}-1} \times 100]$. Robust standard errors in parentheses.
* $p < 0.10$, ** $p < 0.05$, *** $p < 0.01$

Table S11: Doubly Robust Difference-in-Differences Estimator, related to Figure 3.

	(1) OLS	(2) DRDID
National labs × Post Oct. 2014	0.028***	0.024***
	(0.007)	(0.008)
Observations	271,753	271,753

Notes: Column (1) presents OLS estimates of equation (1). Column (2) presents the doubly robust difference-in-differences estimator (Callaway & Zhao, 2020). Explanatory variables in column (2) include the first author country of origin, the number of authors, the number of academic citations and journal dummies. The dependent variable is the number of body-only patent citations. The sample consists of articles published in four journals that

routinely make their articles free to read. Robust standard errors in parentheses.
* $p < 0.10$, ** $p < 0.05$, *** $p < 0.01$
